# Nanofiltration of Multi-Ion Solutions: Quantitative Control of Concentration Polarization and Interpretation by Solution-Diffusion-Electro-Migration Model

**DOI:** 10.3390/membranes11040272

**Published:** 2021-04-08

**Authors:** Marc Fernández de Labastida, Andriy Yaroshchuk

**Affiliations:** 1 Department of Chemical Engineering, Polytechnic University of Catalonia—BarcelonaTech, C/Eduard Maristany 10-14 (Campus Diagonal-Besòs), 08930 Barcelona, Spain; 2 Barcelona Research Center on Multiscale Science and Engineering, C/Eduard Maristany, 10-14 (Campus Diagonal-Besòs), 08930 Barcelona, Spain; 3 Department of Chemical Engineering, Polytechnic University of Catalonia—BarcelonaTech, av. Diagonal 647, 08028 Barcelona, Spain; andriy.yaroshchuk@upc.edu; 4 Catalan Institution for Research and Advanced Studies—ICREA, Passeig Lluís Companys 23, 08010 Barcelona, Spain

**Keywords:** concentration polarization, ion rejection, unstirred-layer thickness, ionic permeance, solution–diffusion–electromigration model

## Abstract

For effective use of advanced engineering models of nanofiltration quality of experimental input is crucial, especially in electrolyte mixtures where simultaneous rejections of various ions may be very different. In particular, this concerns the quantitative control of concentration polarization (CP). This work used a rotating disklike membrane test cell with equally accessible membrane surface, so the CP extent was the same over the membrane surface. This condition, which is not satisfied in the conventional membrane test cell, made possible correcting for CP easily even in multi-ion systems. Ion rejections were studied experimentally for several dominant salts (NaCl, MgCl_2_, Na_2_SO_4_ and MgSO_4_) and trace ions (Na^+^, NH_4_^+^, Cl^−^ and NO_3_^−^) using NF270 membrane. The solution–diffusion–electro–migration model was used to obtain ion permeances from the experimental measurements. The model could well fit the experimental data except in the case of NH_4_^+^. The correlations between the ion permeances and type of dominant salt are discussed in the context of the established mechanisms of NF such as Donnan and dielectric exclusion. The obtained information contributes to the systematic transport characterization of NF membranes and may be ultimately useful for computational fluid dynamics simulations of the performance of the membranes in various applications.

## 1. Introduction

Considerable effort has been devoted to nanofiltration (NF) modelling and several approaches can be found in the literature [1,2,3,4,5,6,7]. Nonetheless, the intricacy of the transport mechanisms of NF complicates the development of predictive models, in particular for multielectrolyte solutions containing both mono and divalent ions. 

In general, there are two kinds of transport models of NF: mechanistic models and irreversible thermodynamics descriptions [1]. The former usually use the concept of nanoporous materials in which ion exclusion (steric, electric and dielectric) and hindered diffusion and convection occur [2,6]. These models are based on macroscopic approaches probably applicable for the description of ultrafiltration but questionable if applied to subnanopores. Besides, they rely on chemical and physical characterization of the membranes that is challenging.

On the other hand, irreversible thermodynamics uses only phenomenological coefficients to describe ion fluxes in terms of gradients of ion electrochemical potentials and transmembrane volume flow. This treatment combined with some assumptions leads to simplified models such as Spiegler–Kedem [4,7], extended Spiegler–Kedem [8], solution-diffusion [9] or solution–diffusion–electromigration [10]. 

Due to the complexity of mechanistic modelling, advanced engineering models based on irreversible thermodynamics seem to remain the approach of choice for practical NF modelling [1]. Even though they do not assume any (microscopic) physical ion exclusion mechanism, they should take into account principal macroscopic physicochemical phenomena using a limited number of fitting parameters obtainable from a well-defined set of experiments. Thus for instance, solution–diffusion–electromigration model (SDEM) of NF accounts for the ion transport due to transmembrane electric fields that spontaneously arise owing to different membrane permeances to cations and anions [10]. This approach has been extensively tested [11,12,13,14,15] and demonstrated to reproduce several observed experimental trends using a limited number of adjustable parameters. 

Including convection to the SDEM ion transport mechanisms would extend the applicability scope of this engineering model. However, this would also double the number of adjustable parameters since the transmission coefficients would be added to the ionic permeances for each ion. The unambiguous determination of this increased number of model parameters relies on the accuracy of the experimental data. An important factor to control is concentration polarization (CP) in membrane test cells.

In a major part of testing devices for pressure membrane processes the extent of CP is inhomogeneous. Disregarding this fact can lead to considerable underestimation or overestimation of the impact of CP depending on the sign of the solute rejection [16]. Therefore, CP inhomogeneity can have a considerable impact on the interpretation of experimental measurements, particularly in multi-ion solutions experiencing very different simultaneous rejections in multi-ion solutions. Meanwhile, in the previous studies using SDEM the thickness of unstirred layer was used as a single adjustable parameter for the description of the rejection of all the solutes (dominant salts as well as trace ions).

To address this issue, a new design of a rotating disklike membrane (RDM) test cell with equally accessible surface was developed [17]. Numerical simulations and experimental validation demonstrated that CP was homogeneously distributed over the entire membrane surface. Once this condition is satisfied, it is easy to decouple the external mass transfer problem from that of transmembrane transport, which is highly desirable considering the complexity of the transport phenomena in NF. In this way, the membrane properties obtained in the RDM cell can further be used as the boundary conditions in computational fluid dynamics (CFD) models describing mass transfer in the spacer-filled channels of the spiral-wound membrane elements or in the feed channels of the tubular or hollow-fiber membranes. 

The set-up was used previously for the CP-correction of the observable ion rejections obtained in NF with a commercial NF270 membrane. However, the intrinsic ion rejections were previously not interpreted by using a model. The aim of this work is to perform such an interpretation by using the SDEM model to investigate the effect of the kind of dominant salt on the ion permeance to the electrolyte mixtures consisting of a dominant salt and trace ions. The results obtained with the RDM cell will be compared with those reported in other studies with the same NF membrane and feed solutions by using a commercial GE SEPA™ CF II test cell.

## 2. Materials and Methods

### 2.1. Materials

Experiments were performed with polyamide thin-film composite NF membrane NF-270 (Dow Chemical Company, Midland, MI, USA). Membranes were mechanically supported by sintered stainless steel disc of 25 mm in diameter and average pore size of 40 µm (GKN Sintered Metals, Bonn, Germany). The chemical reagents used to prepare feed aqueous solutions in the experiments were of analysis grade.

### 2.2. Experimental Set-Up and Operation Procedure

The experimental setup and operation procedures were previously described in [17]. The flat disc membrane employed is 25 mm in diameter although the effective area of filtration is reduced to 2.84 cm^2^ (equivalent to 19 mm in diameter) since in this test cell the membrane filtration in the peripheral membrane zone need to be excluded (see details in [17]). The rotation speed was set to 1000 rpm with a variable frequency drive control in all the experiments and a digital tachometer was used to control it. Feed solution (10 L) was set at a constant temperature of 20 °C ± 1 °C and pumped into the RDM cell running through a filter cartridge (100 μm). After that, concentrated steam was recirculated working in a continuous mode with constant feed composition.

A new piece of membrane was used in each experiment to guarantee the same initial condition in all the measurements. The membranes were soaked in ultrapure water overnight. Before starting an experiment, the membrane was compacted to reach a steady state on the membrane hydraulic resistance avoiding any change during the experiment. First, compaction was done with deionized water at the maximum pressure (15 bar) for 1 h. The water permeability was calculated during the compaction to verify that in the end it reached a steady value. Then, the same procedure was applied thereafter with the feed solution.

Experiments were performed at a constant pump flow rate of 1.5 L/min. The transmembrane pressure was increased from 2 to 14 bar and permeate samples were collected at each point once a steady state was reached, which was controlled by measuring permeate conductivity. Feed samples were also collected initially and at the end of the experiment to check that the feed composition was constant over the experiment.

The experiments were designed to study the effect of a dominant salt on the trace ion rejection. The feed concentration of dominant salts was 0.01 mol/L, whereas for trace salts was 2 × 10^−4^ mol/L in all the experiments.

### 2.3. Analytical Techniques

The conductivity was measured by a conductivity meter (GLP31, Crison, Barcelona, Spain) to have a rough estimate of ion rejection. Afterwards, the samples were analyzed by ionic chromatography (DIONEX ICS-1000/ICS-1100) using two different columns, IONPAC^®^ CS16 and IONPAC^®^ AS23 (Dionex, Sunnyvale, CA, USA), to analyze cations and anions, respectively.

## 3. Theory

### Solution–Diffusion–Electromigration Model

The SDEM is an advanced engineering model based on a (reduced) irreversible thermodynamics approach that describes ion rejections as a function of transmembrane flux using membrane permeances to the ions of the dominant and trace salts as model parameters [10]. 

We use the model of unstirred layer for the description of CP. This model has been proved to be quantitative applicable in the particular case of RDM cell [17], in contrast to any other cell configuration. In this way, the observed rejections measured experimentally corrected for CP as described in [17]. 

The intrinsic rejections estimated for each ion allow obtaining the necessary model parameters to calculate the ionic permeances by means of SDEM. First, the intrinsic rejection of dominant salt, $R_{s}^{int}$, is fitted to obtain the membrane permeance to the dominant salt, *P_s_*:

(1) $$R_{s}^{int}=\frac{\frac{J_{v}}{P_{s}}}{1+\frac{J_{v}}{P_{s}}}\;$$

where $J_{v}$ is the transmembrane flux calculated as:

(2) $$J_{v}=\frac{V_{p}\;}{A\cdot t}$$

where$\;V_{p}$ is the volume of permeate collected, *A* is the membrane active area and t is the sampling time.

Then, intrinsic reciprocal transmissions of trace ions, $f_{t}$, are fitted to Equation (3) as functions of reciprocal intrinsic transmission of dominant salt, $f_{s}$:

(3) $$f_{t}={\left(f_{s}\right)}^{b}+K\cdot \left(\frac{f_{s}-{\left(f_{s}\right)}^{b}}{1-b}\right)$$

where:

(4) $$f_{s}=\frac{1}{1-R_{s}^{int}}$$

(5) $$f_{t}=\frac{1}{1-R_{t}^{int}}$$

$R_{s}^{int}$ and $R_{t}^{int}$ are the intrinsic rejection of dominant salt and trace ion, respectively, *b* and *K* are adjustable parameters.

Finally, permeances to ions of dominant salt ($P_{\pm }$) and permeances to trace ions ($P_{t}$) can be estimated as:

(6) $$P_{\pm }=\frac{P_{s}}{1-\left(\frac{z_{+}}{z_{-}}\right)\cdot b}$$

(7) $$P_{t}=\frac{P_{s}}{K}$$

## 4. Results and Discussion

This section discusses the membrane permeances of ions estimated from a set of experiments using several single dominant salts (NaCl, MgCl_2_, Na_2_SO_4_ or MgSO_4_) and trace salts (NaNO_3_ and/or NH_4_Cl). The intrinsic rejections calculated via the CP correction from the results obtained in a previous work [17] are fitted to the SDEM model to determine membrane permeances to dominant salts and ion permeances in each case. The results are compared with the literature and their consistency and relation with NF separation mechanisms are discussed.

Table 1 presents the membrane permeances to dominant salt and the permeance obtained for each ion from the fitting of experimental data to the SDEM model. It can be seen that there is a notable difference between the experiments where SO_4_^2−^ was the dominant anion with respect to the cases where Cl^−^ was the dominant anion. SO_4_^2−^ is a highly rejected ion, so for Na_2_SO_4_ and MgSO_4_ the membrane permeance to dominant salt is an order of magnitude lower than for the experiments with dominant NaCl and MgCl_2_, which are moderately rejected salts.

**Figure 1:**
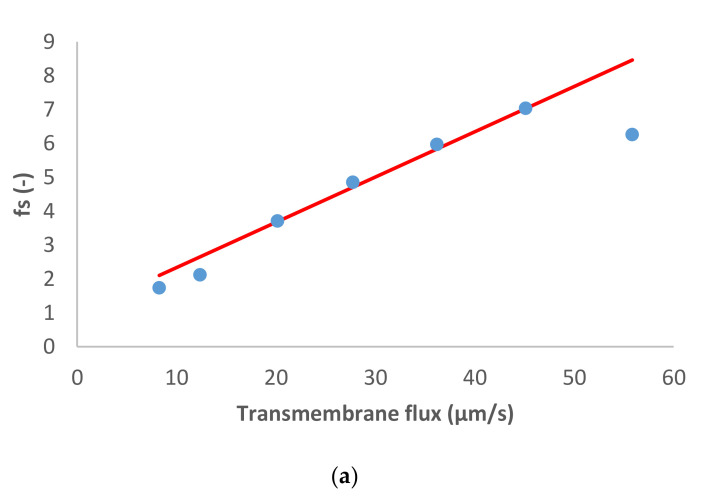

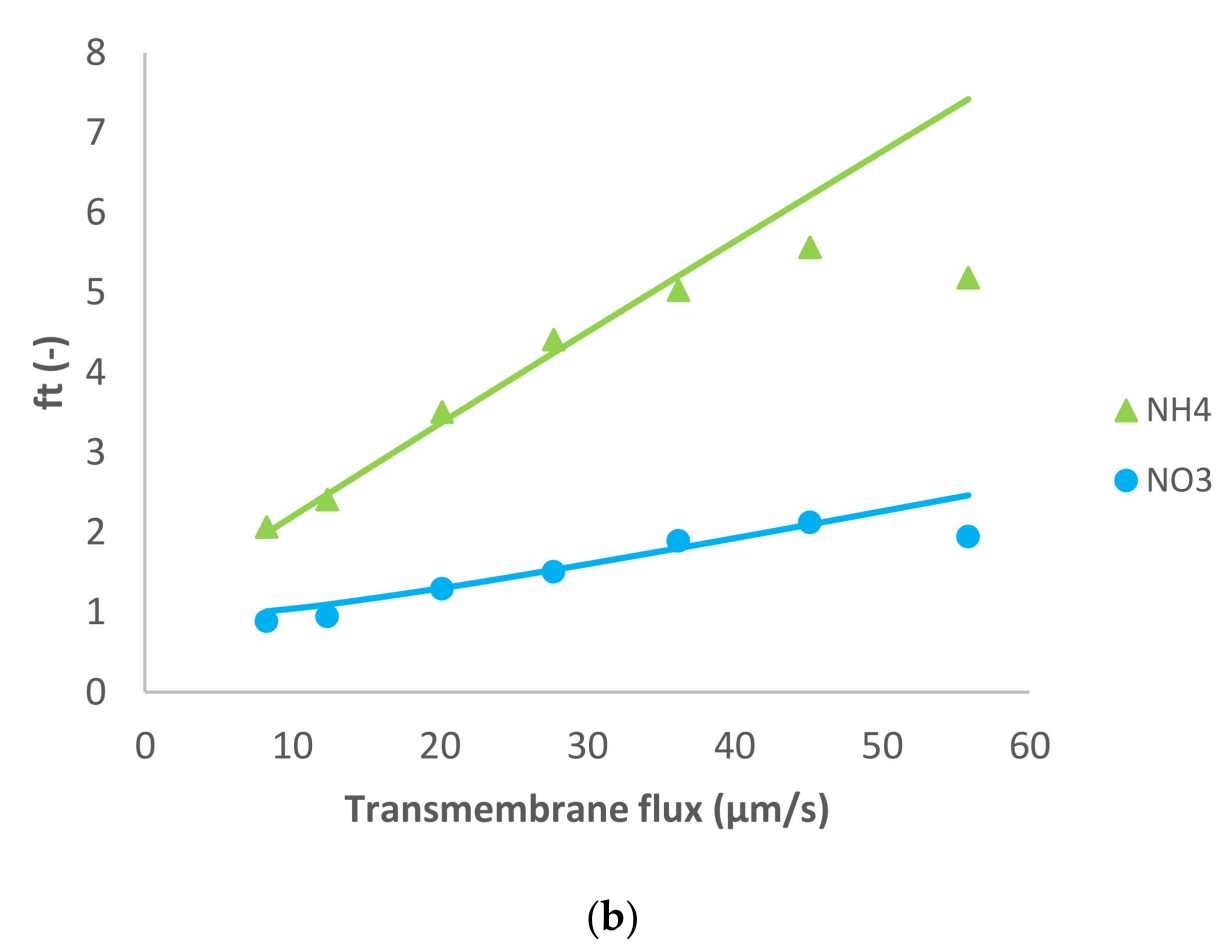
(**a**) NaCl and (**b**) trace ions (${\mathrm{NH}}_{4}^{+}$ and ${\mathrm{NO}}_{3}^{-}$ ) reciprocal intrinsic transmissions dependences on transmembrane flux. The symbols represent experimental data whereas the lines correspond to the solution–diffusion–electromigration model (SDEM) fits.

Figure 1 presents the ion rejection of the dominant salt NaCl and trace ions (NH_4_^+^ and NO_3_^−^) in terms of reciprocal intrinsic transmissions. The symbols represent experimental data whereas the lines correspond to the SDEM fits. The experimental data for the dominant salt are in a relatively good agreement with the model (predicting linear dependence) with exception of the highest transmembrane flux (55 µm/s). A similar situation is observed for the traces, although in the case of NH_4_^+^ the deviations start at a lower transmembrane flux (35 µm/s) and are more pronounced than in the case of the dominant salt. Actually, modelling for NH_4_^+^ became insensitive to the value of ionic permeance as this increased, so it was not possible to estimate an exact value in this case.

Both the dominant salt and the NH_4_^+^ trace were well-rejected as can be seen in Figure 1, their rejections being between 40–80%. On the other hand, NO_3_^−^ trace rejections were below 50% and it even experienced lightly negative values at small transmembrane fluxes. Negative rejections are due to spontaneously arising transmembrane electric fields induced owing to different membrane permeances for the dominant cations and anions [10]. In the case of dominant NaCl solution, the membrane is less permeable to Cl^−^ than to Na^+^ (see Table 1), which is expected given that NF270 is negatively charged. Accordingly, the transport of NO_3_^−^ is enhanced, leading to negative rejections at small transmembrane fluxes due to the fact that the permeance to NO_3_^−^ is noticeably higher than that to Cl^−^. Finally, NO_3_^−^ rejections turn positive when the electromigration NO_3_^−^ flux tends to saturation while the permeate gets ever more diluted due to the linear increase in the transmembrane volume. 

Reig et al. [15] studied the effect of dominant NaCl feed concentration on the removal of NH_4_^+^ and NO_3_^−^ traces. The authors observed that increasing dominant salt concentration decreased the rejection of dominant salt and trace ions. In agreement with this, the membrane permeance to dominant salt determined in the present study (at a lower concentration) is lower than the value reported by Reig et al. 

**Figure 2:**
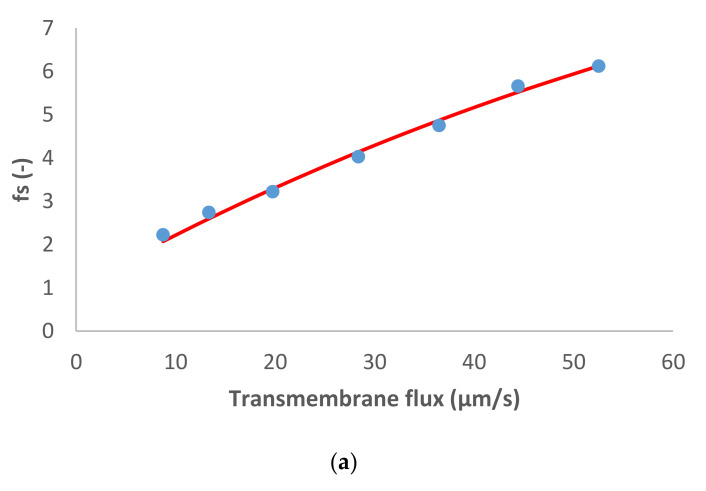

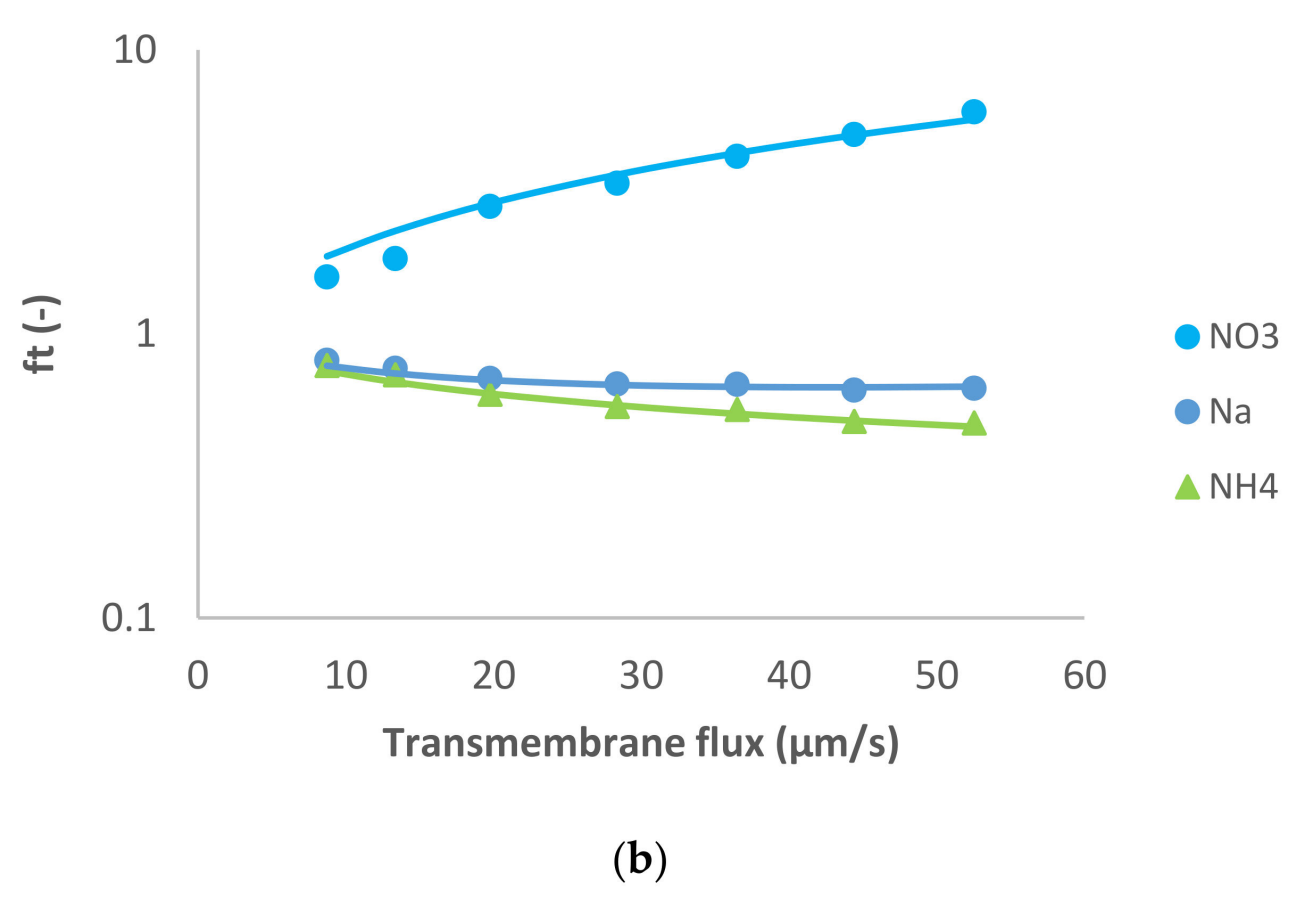
(**a**) MgCl_2_ and (**b**) trace ions (${\mathrm{NO}}_{3}^{-}$, ${\mathrm{Na}}^{+}$, and ${\mathrm{NH}}_{4}^{+}$ ) reciprocal intrinsic transmissions dependences on transmembrane flux. The symbols represent experimental data whereas the lines correspond to the SDEM fits.

The following case study consisted of the dominant salt MgCl_2_ and Na^+^, NH_4_^+^ and NO_3_^−^ as the trace ions. Figure 2 shows the model fit of the experimental data. Similarly to the previous case, for the dominant salt there are some deviations between the experimental data and the modelling curves. However, the quality of the linear fit is quite good.

In this case, the presence of a divalent cation leads to the opposite situation compared with the previous case. The membrane permeance to Mg^2+^ is lower than to Cl^−^ (see Table 1), so the spontaneously arising electric field accelerates the cations. As a result, Na^+^ and NH_4_^+^ presented negative rejections whereas NO_3_^−^ rejection was positive as can be seen in Figure 2.

MgCl_2_ dominant salt rejections are higher than those of NaCl (between 45–90%), which is expectable considering that dielectric exclusion is stronger in electrolytes with double-charge ions. Accordingly, the membrane permeance to MgCl_2_ is lower than to NaCl (Table 1). Due to the better rejection of MgCl_2_ (the larger difference in the permeances between Mg^2+^ and Cl^−^, and much higher ion permeances for both trace cations) in this case the cationic traces experience quite pronounced negative rejections as discussed previously [17]. Similarly to the previous case, for both NH_4_^+^ and Na^+^ the fitted ion rejections were rather insensitive to the assumed values of ion permeances so these could only be determined by the order of magnitude. Nonetheless, they are clearly by an order of magnitude larger than in the case of dominant NaCl. This may be due to a much stronger exclusion of Mg^2+^ ions from the membrane phase than that of Cl^−^. As demonstrated in [18] such preferential exclusion of cations gives rise to the appearance of an interphase electrostatic-potential difference that can enhance the partitioning of other cations (including monovalent) into the membrane phase and can considerably increase the membrane permeance to them. 

On the other hand, NO_3_^−^ was much better rejected than in the presence of NaCl as the dominant salt (intrinsic rejections were up to 85%), which is expected in view of the fact that the electric field decreases the passage of NO_3_^−^ through the membrane when MgCl_2_ is the dominant salt. 

Pagès et al. [14] studied MgCl_2_ as a dominant salt and a number of trace ions using a higher feed solution concentration (0.1 M) than the one used in this work. The authors reported a membrane permeance to dominant salt around 2 µm/s, which is lower than the one estimated in this work at a lower feed solution concentration. Unlike the case of dominant NaCl, this trend is in disagreement with the simple Donnan rejection mechanism but has already been reported in other studies for MgCl_2_ [19] as well as for CaCl_2_ [3]. A possible explanation for this as a result of combination of impact of unequal ion exclusion from the membrane and its negative surface charge has been put forward in [20].

The last two cases (Na_2_SO_4_ and MgSO_4_) have in common the divalent anion SO_4_^2−^. The membrane permeance to this ion is much lower than the permeance to either of the dominant cations (see Table 1). Therefore, in both cases the transport of trace anions is improved whereas that of trace cations is retarded. In these cases, trace ions cannot be accounted as authentic traces due to the very high rejections of SO_4_^2−^. Accordingly, the reciprocal transmission of the more permeable ion (such as Na^+^ in Na_2_SO_4_) could be basically lower than that for SO_4_^2−^ since the transmembrane passing of anion traces partially contributed to the fulfilment of zero electric current condition.

**Figure 3:**
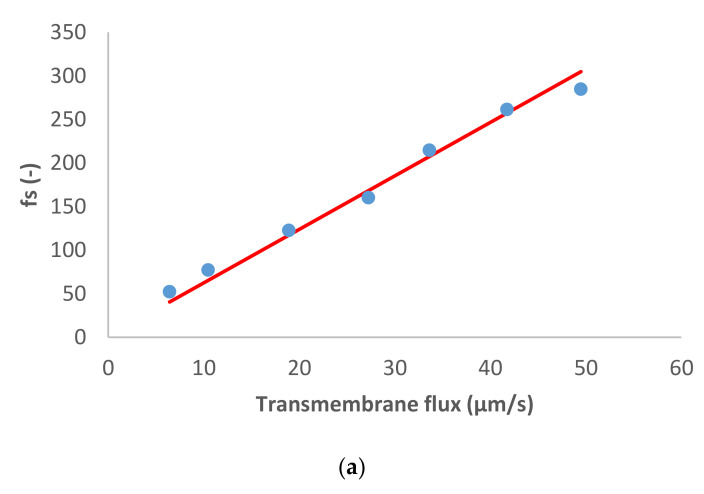

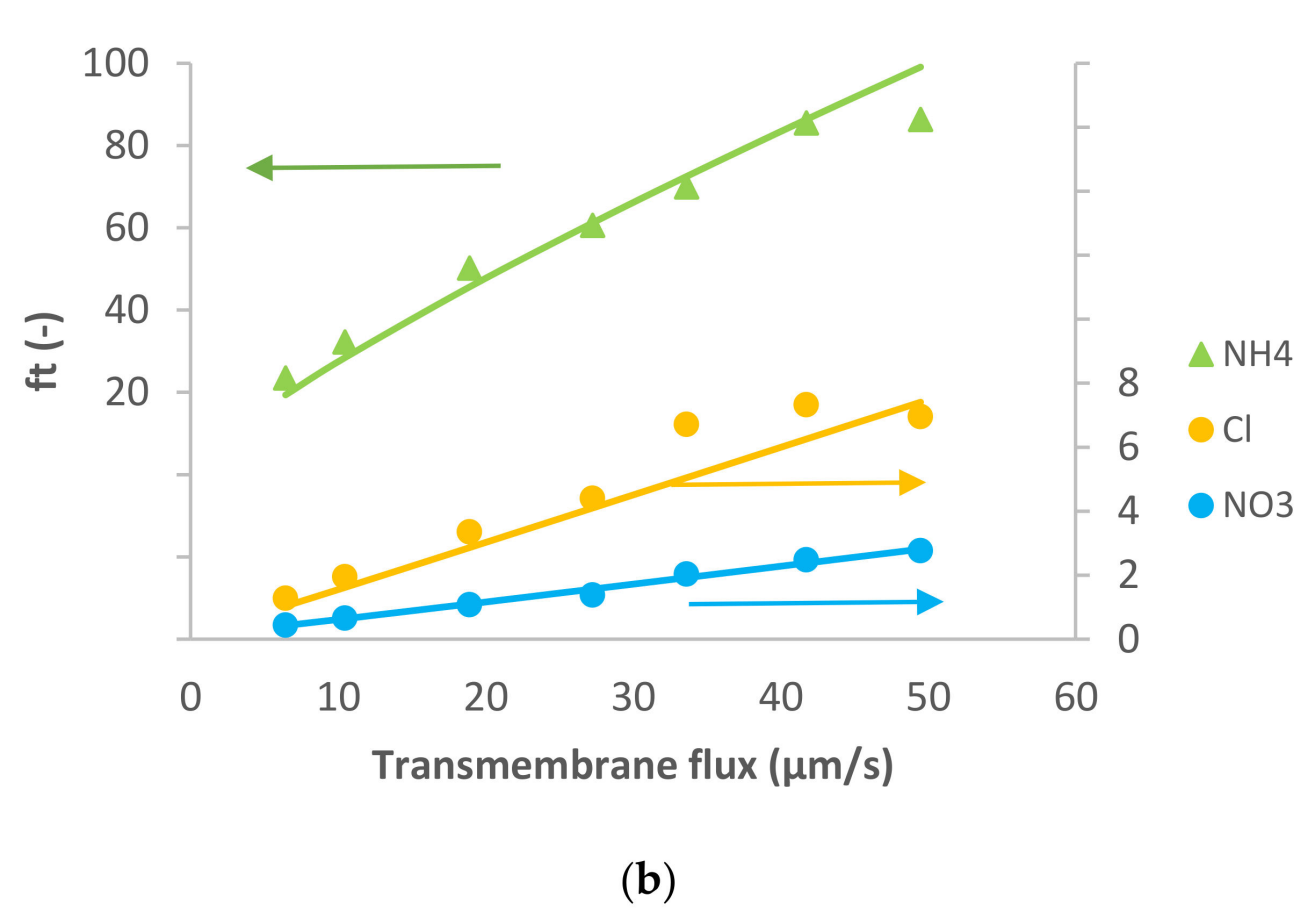
(**a**) Na_2_SO_4_ and (**b**) trace ions (${\mathrm{NH}}_{4}^{+}$, ${\mathrm{Cl}}^{-}$ and ${\mathrm{NO}}_{3}^{-}$ ) reciprocal intrinsic transmissions dependences on transmembrane flux. Main axis corresponds to reciprocal intrinsic transmission for ${\mathrm{NH}}_{4}^{+}$, whereas the secondary axes show the values for ${\mathrm{Cl}}^{-}$ and ${\mathrm{NO}}_{3}^{-}$. The symbols represent experimental data whereas the lines correspond to the SDEM fits.

Figure 3 shows the results obtained in the case of dominant Na_2_SO_4_ and traces of NH_4_^+^, Cl^−^ and NO_3_^−^. Notably, in situations of a very strong asymmetry in the permeances to the dominant cation and anions (like in the case of Na_2_SO_4_), the determination of permeance of the more permeable ion (such as Na^+^ in Na_2_SO_4_) becomes very imprecise since its variation does not influence the salt permeance. Accordingly, the value of Na^+^ permeance in this case is just orientative. The situation with NH_4_^+^ is similar.

The dominant salt was highly rejected as well as the NH_4_^+^ trace. Concerning the trace anions, a different behavior was observed between the two traces: NO_3_^−^ presented initially pronounced negative rejections (as much as 131%) that turned to positive reaching a maximum value of 63% while Cl^−^ rejections were positive in all the transmembrane flux range studied (between 21–85%). 

Such behavior of trace NO_3_^−^ is primarily explained by the very high rejection of dominant salt (and very strong asymmetry in the permeances to the dominant cations and anions). Due to these factors, the spontaneously arising electric fields are very strong already at quite small transmembrane volume flows (hence the initially pronounced negative rejections). At the same time, these fields tend to become saturated at relatively low fluxes, which gives rise to the rapid change to the positive rejections of trace NO_3_^−^. The comparison of the behavior of traces of NO_3_^−^ and Cl^−^ in this case shows that occurrence of negative rejections also requires the trace anion to have a certain minimum permeance, which is largely surpassed by NO_3_^−^ but not reached by Cl^−^ (hence, the lack of negative rejections for it).

**Figure 4:**
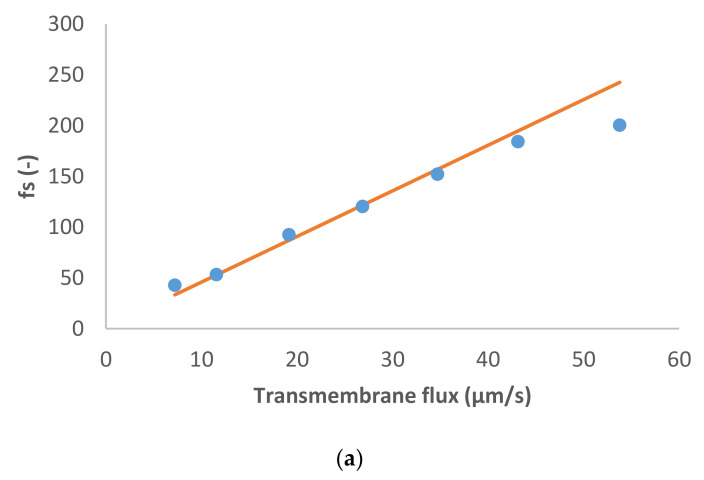

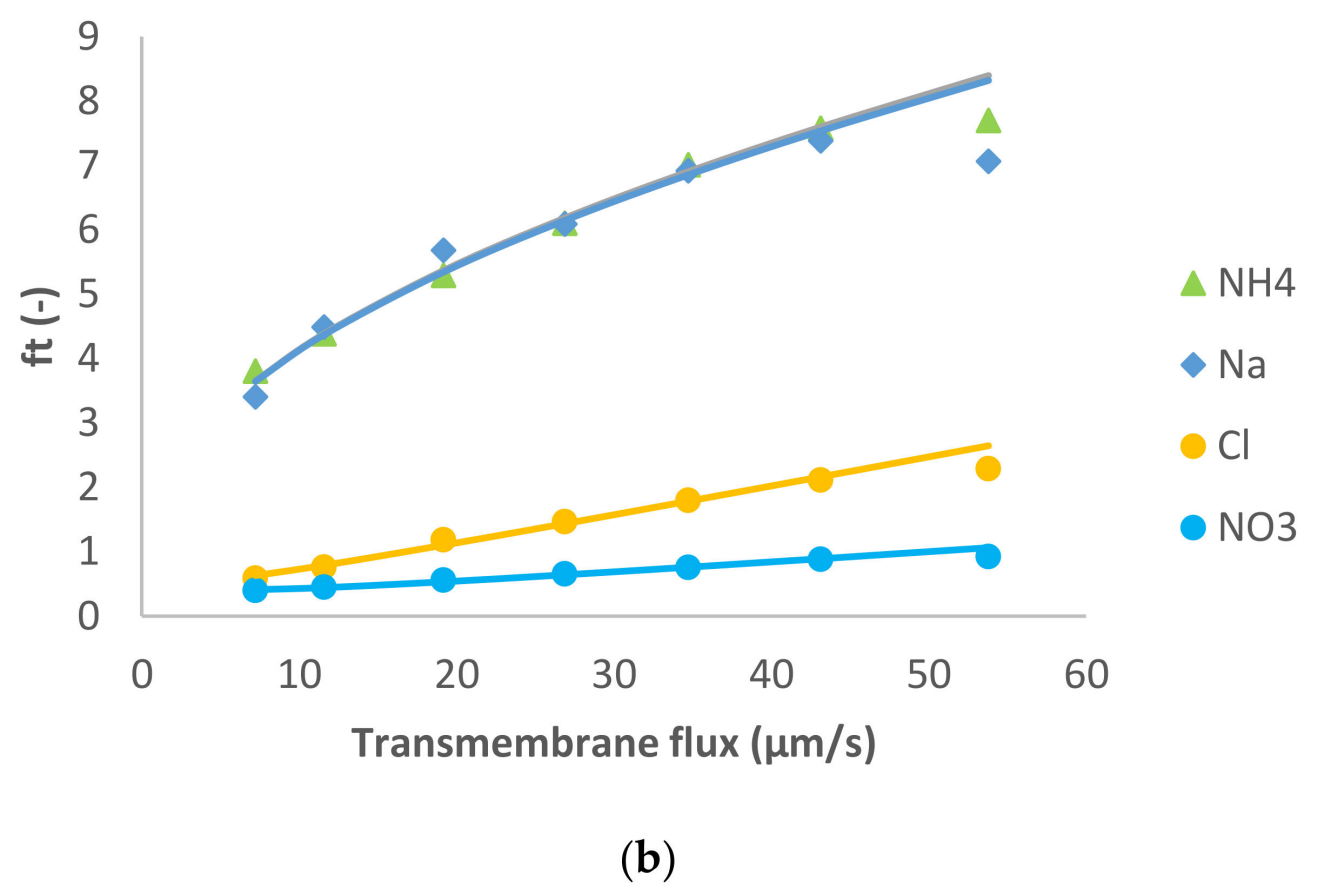
(**a**) MgSO_4_ and (**b**) trace ions (${\mathrm{NH}}_{4}^{+}$, ${\mathrm{Na}}^{+}$, ${\mathrm{Cl}}^{-}$ and ${\mathrm{NO}}_{3}^{-}$ ) reciprocal intrinsic transmissions dependences on transmembrane flux. The symbols represent experimental data whereas the lines correspond to the SDEM fits.

The last case studied is MgSO_4_ like dominant salt and NH_4_^+^, Na^+^, Cl^−^ and NO_3_^−^ as trace ions (Figure 4). The dominant salt was highly rejected, as in the previous case, and the trace cations were also rejected. Both trace anions exhibited negative rejections in this case: Cl^−^ was negatively rejected at small transmembrane flux (−70%), but its rejection increased up to 60%, while the values for NO_3_^−^ were negative in all the range of transmembrane fluxes studied (from −151% to −5%). To fit these more pronounced negative rejections by the model we had to assume noticeably higher ion permeances for the trace single-charge anions than in the case of dominant Na_2_SO_4_. Remarkably, the ratio of permeances to NO_3_^−^ and Cl^−^ for all the studied cases was roughly around 3.

The obtained sequence of membrane permeances to dominant salt is in qualitative agreement with the published results for negatively charged membranes [14,21]. It can be explained by a combination of Donnan and dielectric exclusion of ions. Donnan exclusion is due to the interactions of ions with fixed electric charges. Dielectric exclusion is caused by interactions between charged solutes and bound charges induced by them at pore surfaces due to the different dielectric constants of the membrane matrix and the liquid inside the pores [22]. Donnan exclusion is stronger for double-charge coions (ions whose charge sign coincide with that of fixed charges) while dielectric exclusion is much stronger for double-charge ions irrespective of the sign of their charge. This explains the much lower membrane permeance to Na_2_SO_4_ than to NaCl. Dielectric exclusion alone would give rise to a still stronger exclusion of MgSO_4_ than of Na_2_SO_4_. However, the double-charged Mg^2+^ may well strongly bind to the negative surface charge thus reducing its magnitude and that of the Donnan exclusion as pointed out by Freger et al. [3] and recently developed by Freger [23]. Likely for that reason there were deviations from Donnan and dielectric exclusion mechanisms and the membrane permeance to MgSO_4_ was somewhat higher than to Na_2_SO_4_.

Na^+^ and Cl^−^ were studied both as dominant and trace ions. In the cases where Na^+^ was part of a dominant salt (NaCl, Na_2_SO_4_) lower values of permeances were obtained. The lowest permeance to Na^+^ corresponds to Na_2_SO_4_, which is expected given that it is strongly affected by dielectric exclusion in this case. Indeed, the interphase potential attracting sulfate ions to the membrane phase (to make their concentration stoichiometric to that of Na^+^) simultaneously expulses positively charged Na^+^ ions [18]. In the case of dominant MgCl_2_, the situation is the opposite, namely, the interphase potential (arising due to the different extents of dielectric exclusion for Mg^2+^ and Cl^−^) attracts cations to the membrane phase in this case making the permeance to Na^+^ ions high. Finally, in the case of MgSO_4_ the extent of dielectric exclusion for both dominant ions is roughly the same so the additional trace Na^+^ attraction to the membrane phase does not occur and the permeance to it is lower, accordingly.

Regarding Cl^−^, the highest permeance was obtained for it when added as a trace to dominant MgSO_4_. This could be explained by the presence of a divalent cation, which reduced the Donnan exclusion of anions (see above). Qualitatively the same occurrence could explain the relatively large permeance to Cl^−^ as a part of dominant MgCl_2_. The Cl^−^ permeances are surprisingly close in the cases of dominant NaCl and Na_2_SO_4_. Given the very different patterns of interaction of dominant anions with the membrane in these two cases, this seems to be rather a result of the accidental compensation of counteracting trends characteristic of Donnan and dielectric exclusion.

NH_4_^+^ and NO_3_^−^ were trace ions in all the experiments. For the NH_4_^+^ traces, the fitted permeances in most cases were so high that the rejections became insensitive to the permeance to this ion. Accordingly, only the lower limits for the permeances could be determined. Just for the dominant MgSO_4_ the fitting procedure allowed to estimate a specific value for the NH_4_^+^ permeance. Even though the permeances could not be determined accurately, the results seem to indicate that the highest permeance occurred in MgCl_2_, which could have the same mechanisms as in the case of traces of Na^+^ (see above). In the case of dominant Na_2_SO_4_, the asymmetry of the dielectric exclusion of the dominant cations and anions was in favor of the latter. Accordingly, the interphase potential repulsed cations (including traces of NH_4_^+^) from the membrane phase, hence the lower permeance. Overall, the permeances of NH_4_^+^ seem to be noticeably larger than those of Na^+^, which is rather difficult to explain by simple mechanisms given, for example, the very close hydrated radii of those two ions in aqueous solutions. Apparently, some more subtle phenomena related to different details of the interaction of these two ions with water and membrane matrix are in play.

The dependence of the permeances of NO_3_^−^ on the kind of dominant salt roughly followed that observed for Cl^−^ but the NO_3_^−^ permeances on average were around three times larger. Again, this is rather difficult to explain by simple mechanisms given the very close hydrated radii of those two ions in aqueous solutions. As in the case of NH_4_^+^ vs. Na^+^ subtler mechanisms seem to be in play.

## 5. Conclusions

Modelling NF processes is a difficult task due to the complexity of transport phenomena. CP inhomogeneity over the membrane surface complicates the task additionally. Therefore, the unambiguous determination of model parameters from experimental data requires quantitative control of CP. The use of a test cell that satisfies this condition may provide the quality input required in the modelling of the performance of practical membrane modules.

The effect of the valence type of the dominant salt on the rejection of single-charge trace cations and anions was studied using the RDM cell with an equally accessible membrane surface developed previously. In this way, the CP extent was the same over the whole membrane surface.

SDEM allowed fitting experimental data and determining membrane permeances to ions except for trace NH_4_^+^. In that case, the ion rejection became insensitive to the exact values so the permeance could only be estimated in one of the studied cases (dominant MgSO_4_). 

The results obtained in terms of membrane permeances to the dominant salts are in qualitative agreement with those obtained in other studies with the same NF membrane and feed solutions by using the GE SEPA™ CF II test cell. 

It was confirmed once again that the differences between the membrane permeances to the dominant salt ions give rise to spontaneously arising electric fields that strongly influence the trace ion rejections. Due to the action of those fields, the ion rejections are not directly correlated with the ion permeance in most cases. At the same time, they can be quantitatively related to them by using the SDEM model.

## Figures and Tables

**Table 1:** Membrane permeance to dominant salt and membrane permeance to each ion depending on dominant salt.

Dominant Salt	Ps (µm/s)	Ion Permeance (µm/s)					
		Na^+^	Mg^2+^	NH_4_^+^	Cl^−^	NO_3_^−^	SO_4_^2−^
NaCl	7.5	20–113	-	>60	3.9	13.8	-
MgCl_2_	6.1	>250	3.7	>700	9.3	16.9	-
Na_2_SO_4_	0.16	>0.8	-	>100	3.4	9.8	0.06
MgSO_4_	0.23	79.4	0.9	80	15.7	41.9	0.13

## List of Symbols

*A*	Membrane active area (m^2^)
*b*	Model parameter (-)
$f_{s}$	Intrinsic reciprocal transmission of dominant salt (-)
$f_{t}$	Intrinsic reciprocal transmission of trace ion (-)
$J_{V}$	Transmembrane flux (µm/s)
*K*	Model parameter (-)
$P_{s}$	permeance to dominant salt (µm/s)
$P_{+}\;$	Permeance to ions of dominant salt (µm/s)
$P_{t}\;$	Permeance to trace ions (µm/s)
$R_{s}^{\left(obs\right)}$	Observed rejection of the salt (-)
$R_{s}^{\left(int\right)}$	Intrinsic rejection of the salt (-)
$R_{t}^{\left(int\right)}$	Intrinsic rejection of the trace ion (-)
t	Sampling time (s)
$V_{p}$	Volume of permeate collected (m^3^)
$Z_{i}$	Charge of ion (-)
